# Antiperspirant effects and mechanism investigation of Mulisan decoction in rats based on plasma metabolomics

**DOI:** 10.1080/13880209.2022.2074465

**Published:** 2022-05-28

**Authors:** Shan-Peng Ma, Wei-Ping Ma, Shi-Ning Yin, Xiang-Yue Chen, Xiao-Qing Ma, Bao-Hong Wei, Jing-Guang Lu, Hong-Bing Liu

**Affiliations:** a Key Laboratory of Marine Drugs, School of Medicine and Pharmacy, Ocean University of China, Qingdao, China; b Qingdao Institute for Food and Drug Control, Qingdao, China; c NMPA Key Laboratory for Quality Research and Evaluation of Traditional Marine Chinese Medicine, Qingdao, China; d Laboratory for Marine Drugs and Bioproducts, Pilot National Laboratory for Marine Science and Technology, Qingdao, China

**Keywords:** Traditional Chinese medicine, hyperhidrosis, pharmacodynamics, metabolic mechanism

## Abstract

**Context:**

Mulisan decoction (MLS) is a classic formula of traditional Chinese medicine for treating hyperhidrosis. The mechanism remains unclear.

**Objective:**

To investigate the antiperspirant effect and underlying mechanisms of MLS.

**Materials and methods:**

Fifty rats were divided into control, model, and three doses of MLS intervention groups (*n* = 10). Rats except for control group were induced diseases features of the applicable scope of MLS *via* i.p. reserpine (0.5 mg/kg/d) for 10 days. From day 11, MLS groups were administrated orally MLS at 0.6, 3, and 15 g/kg once a day for 14 days, respectively. After the last administration, sweating was induced in all rats *via* s.c. pilocarpine (25 mg/kg), the right hind foot of rats was stained, and sweat point numbers were observed. Rat serum was collected to detect IL-2, IL-6, IFN-γ, and TNF-α. Rat plasma was collected for endogenous metabolite analysis *via* UPLC-QE-Focus-MS.

**Results:**

Rats treated with MLS presented a significant decrease in sweat point numbers (13.5%), increase in body weight (13.2%), and promotion in the balance of Th1/Th2 cytokine ratio *via* increasing IL-2 (38.3%), IFN-γ (20.1%), and TNF-α (22.0%) and decreasing IL-6 (24.7%) compared with the model group (*p* < 0.05). Plasma metabolomics disclosed 15 potential biomarkers related to model rats, of which two could be significantly reversed by MLS (*p* < 0.05). The involved pathways were pantothenate and CoA biosynthesis, and porphyrin metabolism.

**Conclusions:**

MLS demonstrated a good antiperspirant effect and metabolism improvement. These findings inspire more clinical study validation on immune improvement and antiperspirant effect.

## Introduction

Hyperhidrosis is a symptom characterised by a general or local phenomenon of increased sweat, mainly caused by physiology or pathology. The aetiology of pathological hyperhidrosis is complex, usually observed in some diseases such as Parkinson's disease, anxiety neurosis, and various body injuries (Ohshima and Tamada 2016; van Wamelen et al. 2019). Th1/Th2 cytokine balance has been reported to be involved in the development and treatment of hyperhidrosis. Yang (2016) pointed out that the switch from Th1 to Th2 in pregnant women could lead to the deterioration of sweat glands function related diseases such as hidradenitis and hyperhidrosis. Wang et al. (2020) found that TNF-α can inhibit the differentiation of mesenchymal stromal cells into sweat gland cells, which may have a great impact on the sweating state.

At present, there is no effective therapeutic drug for sweating in modern medicine. As a traditional Chinese medicine (TCM) formula, Mulisan decoction (MLS) has been used clinically for nearly 1000 years and has achieved an excellent curative effect on sweating symptom caused by various diseases (Zhou et al. 2017; Hu 2021). For example, Xu (2018) used MLS to treat hyperhidrosis of Parkinson's disease and achieved a total effective rate of 80%. Chen (2017) used MLS to treat primary palmar hyperhidrosis and reached a real effective rate of 94.7%. Lin and Fu (2011) used MLS to treat night sweat after transcatheter arterial chemoembolization for primary liver cancer, and the total effective rate was 94.2%.

MLS is recorded in the Chinese herbal ancient book ‘Taiping Huimin Heji Jufang’ in A.D. 1148 and comprises four medicinal slices: Ostreae Concha [calcined shell of *Ostrea gigas* Thunberg (Ostreae), ‘Duan-mu-li’ in Chinese], Astragali Radix [radix of *Astragalus membranaceus* (Fisch.) Ege. var. *mongholicus* (Ege.) Hsiao (Leguminosae), ‘Huang-qi’ in Chinese], Ephedrae Radix et Rhizoma [radix and rhizome of *Ephedra sinica* Stapf (Ephedraceae), ‘Ma-huang-gen’ in Chinese], and Tritici Levis Fructus [floating fruit of *Triticium aestium* L. (Gramineae), ‘Fu-xiao-mai’ in Chinese]. Our previous work has done a chemical investigation on the aqueous extract of MLS and identified flavonoids including formononetin, calycosin, ononin, calycosin-7-*O*-β-d-glucopyranoside, methylnissolin, 9,10-dimethoxypterocarpan-3-*O*-β-d-glucoside, isomucronulatol, and isomucronulatol-7-*O*-β-d-glucoside (Zhao et al. 2019), as well as nitrogen-containing compounds including guanosine, adenosine, 2′-deoxyadenosine, zarzissine, and tryptophan (Chen 2020).

Although MLS has sound antiperspirant effects in the clinic, the possible mechanism of its treatment is still unclear. In this study, we selected a reserpine-induced rat as an animal model to further study the antiperspirant mechanism of MLS. As a typical therapeutic drug for hypertension, reserpine can cause the synthesis and secretion of serotonin, norepinephrine, epinephrine, and dopamine, and then induce some peripheral sympathetic symptoms, such as depression, fatigue, weakness, loss of appetite, and diarrhoea (Antkiewicz-Michaluk et al. 2015). Thus, reserpine is often used in scientific research to induce the animal model of Parkinson's, depression, tension, spleen deficiency, and other diseases (Khurana and Bansal 2019; Leal et al. 2019; Zheng et al. 2020), which is consistent with the applicable scope of MLS. Based on intraperitoneal injection of reserpine, we induced spontaneous sweating symptoms by intraperitoneal injection of pilocarpine. The established rat model was used to evaluate the antiperspirant effect of MLS on sweat point numbers, body weight, and the Th1/Th2 ratio. Meanwhile, we tried to determine the antiperspirant mechanism of MLS by screening potential endogenous metabolite biomarkers and forecasting possible pathways based on the plasma metabolomics combined with KEGG pathway enrichment analysis. As a result, this study provided new insights and the basis for the clinical application of MLS.

## Materials and methods

### Medicinal materials

The medicinal slices, Ostreae Concha (calcined shell of *Ostrea gigas*, L/N: 160701, produced in Shandong province, China), Astragali Radix (radix of *Astragalus membranaceus* var. *mongholicus*, L/N: 170501, produced in Shanxi province, China), Ephedrae Radix et Rhizoma (radix and rhizome of *Ephedra sinica*, L/N: 161101, produced in Ningxia province, China) were provided by Pharmacy of the Affiliated Hospital of Qingdao University. Tritici Levis Fructus (floating fruit of *Triticium aestium*, L/N: 180124, produced in Shandong province, China) were purchased from Nanjing Haiyuan TCM decoction pieces Co., Ltd. (Nanjing, Jiangsu, China). All medicinal slices were authenticated by Professor Fengqin Zhou from Shandong University of Traditional Chinese Medicine (Ji’nan, China) according to the Pharmacopoeia of the People's Republic of China identification key (2020, Volume 1). Voucher specimens were deposited at the laboratory of marine TCM, Ocean University of China (Qingdao, China).

The preparation of MLS was decocted following the traditional method. In brief, 1 kg of mixed medicinal slices (Ostreae Concha, Astragali Radix, Ephedrae Radix et Rhizoma, and Tritici Levis Fructus, 6:6:6:5) was soaked in 12 L distilled water for 30 min, then decocted (100 °C) for 1.5 h and repeated once. The combined water extracts were concentrated and dried under reduced pressure to obtain the MSL extract. The extracted dried powder (1 g) was equivalent to 8.40 g material herbs.

### Reagents

Pilocarpine eye drops (L/N: 18041301) were purchased from Shandong Boshilun Furuida Pharmaceutical Co., Ltd. (Ji’nan, Shandong, China). Reserpine injection (L/N: 1705091) was purchased from Tianjin Jinyao Pharmaceutical Co., Ltd. (Tianjin, China). Urethane (L/N: 20150108) was purchased from Shanghai Shanpu Chemical Co., Ltd. (Shanghai, China). Rat ELISA kit interleukin 2 (IL-2, L/N: JYM0648Ra), interleukin 6 (IL-6, L/N: JYM0646Ra), tumour necrosis factor α (TNF-α, L/N: JYM0635Ra), and γ-interferon (IFN-γ, L/N: JYM0654Ra) were all purchased from Wuhan Jiyinmei Technology Co., Ltd. (Wuhan, Hubei, China). Hotan Takagaki reagent was self-made by our laboratory. Hotan Takagaki reagent A was prepared with iodine and absolute ethanol in the ratio of 1:50 (w/v). Hotan Takagaki reagent B was prepared with soluble starch and peanut oil in the ratio of 1:2 (w/v).

### Model establishment and grouping

Fifty specific pathogen free male Sprague-Dawley rats (200 ± 20 g) were purchased from Ji’nan Pengyue experimental animal breeding Co., Ltd. (Ji’nan, Shandong, China). After adaption for one week, rats were randomly divided into 5 groups according to body weight: control group (Control, *n* = 10), RP model group (Model, *n* = 10), low dose of MLS group (0.6 g/kg, *n* = 10), medium dose of MLS group (3 g/kg, *n* = 10), and high dose of MLS group (15 g/kg, *n* = 10). Except for the control group, rats were established by intraperitoneal injection with reserpine (0.5 mg/kg/d) for 10 days. Low, medium, and high dose group rats were administrated orally MLS at doses of 0.6, 3, and 15 g/kg once a day for 14 days, respectively. The control and model group rats were given the same volume of water. The body weight of rats was measured every day. Animal procedures were approved by the Institutional Animal Care and Use Committee of the Ocean University of China (OUC-SMP-2019-06-01).

### Sweat point numbers observation

After the last administration, rats in all groups were subcutaneously injected with pilocarpine (25 mg/kg). After 15 min, the right hind foot of all rats was cleaned with absolute ethanol and coated with Hotan Takagaki reagent A. After desiccation, the right foot was coated with Hotan Takagaki reagent B and stained for 15 min. The sweat points in the right foot of each group were observed and counted with a 10-fold magnifying glass.

### Serum cytokines detection

After fasting for 24 h at the end of the experiment, rats were anaesthetised by intraperitoneal injection with 20% urethane (0.80 g/kg). The blood (2 mL) was collected in the abdominal aortic blood of all rats, stood for 30 min. The serum was obtained by centrifugation of blood for 10 min (3000 rpm, 4 °C). Serum IL-2, IL-6, IFN-γ, and TNF-α were detected using ELISA kits according to the instructions. The absorbance of the solution was measured with a microplate reader (PA, USA) at 450 nm.

### Organ index calculation

After taking blood, the rats were euthanized, and the spleen and thymus were taken and accurately weighed. The spleen and thymus indices were calculated according to the formula: Organ index = Organ weight (g)/Body Weight (g) × 100%.

### Plasma metabolomics analysis

#### Metabolomics sample pre-treatment

The plasma of each group was collected by centrifugation of blood obtained from the abdominal aortic for 10 min (3000 rpm, 4 °C). Plasma sample (200 μL) was added with 800 μL methanol and mixed. After centrifugation for 15 min (12,000 rpm, 4 °C), the supernatant (200 μL) was dried by nitrogen air flow and re-dissolved with 200 μL of 80% methanol (v/v). The supernatant was obtained for analysis after centrifugation for 15 min (12,000 rpm, 4 °C). The same amount of each sample was taken and mixed well as quality control (QC) sample for repeated injection during sample analysis to detect the stability of the instrument.

#### UPLC-MS/MS conditions

Chromatographic experiments were performed on a pre-column of Agilent InfinityLab Poroshell 120 (2.1 × 5 mm, 2.7 μm) and a column of InfinityLab Poroshell 120 EC-C18 (2.1 × 150 mm, 2.7 μm) using a Thermo UltiMate 3000 UPLC^TM^ system (Thermo Fisher Scientific, USA). The column temperature was set at 35 °C with an injection volume of 1 μL, and the mobile phase consisted of solvent A (0.1% formic acid in water) and solvent B (acetonitrile) at a flow rate of 0.2 mL/min. The gradient elution procedure was as follows: 0–3 min, 5–5% B; 3–5 min, 5–55% B; 5–12 min, 55–100% B; 12–20 min, 100–5% B; 20–22 min, 5–5% B.

Mass spectrometry was performed on a Q Exactive™ Focus Orbitrap™ (Thermo Fisher Scientific, USA). The instrument was operated using a HESI source in positive and negative mode. The sheath gas volume flow was set at 35 psi, and the auxiliary gas volume flow was 10 psi. The spray voltage was 3 kV. The capillary temperature was 350 °C, and the auxiliary gas temperature was 300 °C. The quality scanning range was 70–1500 m/z, and the detection resolution was 70000. The secondary mass spectrometry adopted high energy collision-induced dissociation with 30 eV ionisation energy and 17,500 resolutions.

### Data analysis strategy

Raw data were uploaded to Progenesis QI Version 2.0 software (Waters, USA) for baseline filtering, peak identification, peak alignment, and normalisation to obtain a two-dimensional data matrix composed of retention time, *m/z*, and ionic intensity of metabolite. The processed data matrix was imported into Simca-p 14.0 for multivariate statistical analysis. Firstly, unsupervised principal component analysis (PCA) was used to observe the sample separation and eliminate abnormal samples. The supervised orthogonal partial least squares discrimination analysis (OPLS-DA) was used to predict the components with variable importance in the project (VIP) greater than 1 between control and model groups, and the components with VIP > 1 and *p* < 0.05 of *t*-test were screened and putatively identified through the database (KEGG, HMDB, lipid maps, massbank, etc.). The screened potential biomarkers were introduced into the metabolic pathway analysis online platform (https://www.metaboanalyst.ca/faces/home.xhtml) for seeking metabolic pathways related to RP rats. Based on this, the regulatory effect of MLS on RP rats was evaluated.

### Statistical analysis

The experimental data were expressed with mean ± standard deviation. The difference between groups was compared using one-way ANOVA with the Bonferroni method, and *p* < 0.05 was considered a significant difference.

## Results

### Effects of MLS on body weight, organ index, and sweat points of RP rats

**Figure 1.**
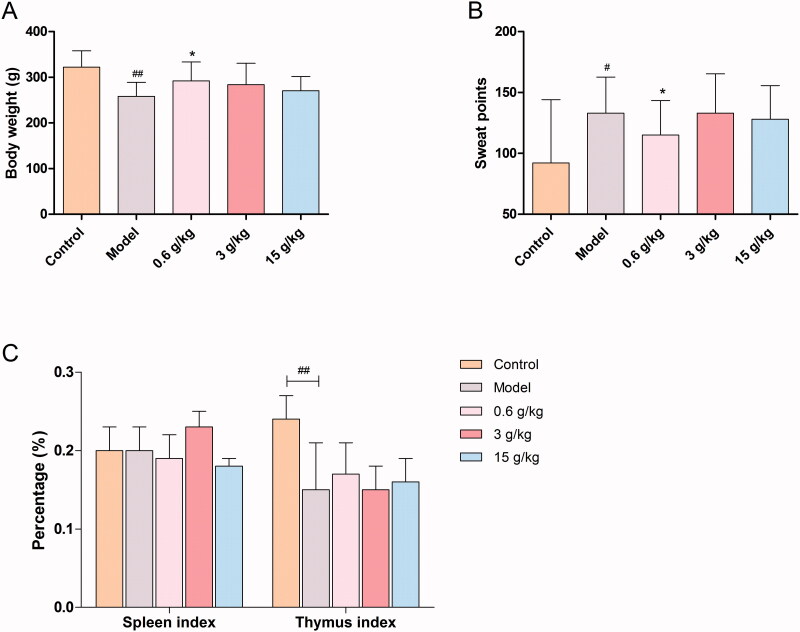
Effects of Mulisan decoction (MLS) on the levels of (A) body weight, (B) sweat points, and (C) organ index in reserpine and pilocarpine (RP) rats (*n* = 10). ^#^*p* < 0.05 and ^##^*p* < 0.01 when compared with the control group; *p < 0.05 and **p < 0.01 when compared with the model group.

Compared with the control group, the body weight decreased significantly in the model group (*p* < 0.01), showing the symptom of spleen deficiency, which proved the successful establishment of the model. Compared with the model group, the body weight in each administration group showed an increasing trend, especially, body weight in the low dose group increased significantly (*p* < 0.05) (Figure 1(A)).

Compared with the control group, the sweat points of the model group increased significantly (*p* < 0.05). Compared with the model group, the sweat points showed a significant decrease in the low dose group (*p* < 0.05), proving that low dose MLS had an antiperspirant effect on RP rats (Figure 1(B)). Compared with the control group, the spleen index in the model group showed no significant change, while the thymus index decreased significantly. Compared with the model group, the thymus index showed an upward trend in each administration group, most obviously observed in the low dose group, suggesting that low dose MLS had a regulatory effect on cell immunity (Figure 1(C)).

### Effects of MLS on the levels of serum cytokines in RP rats

**Figure 2.**
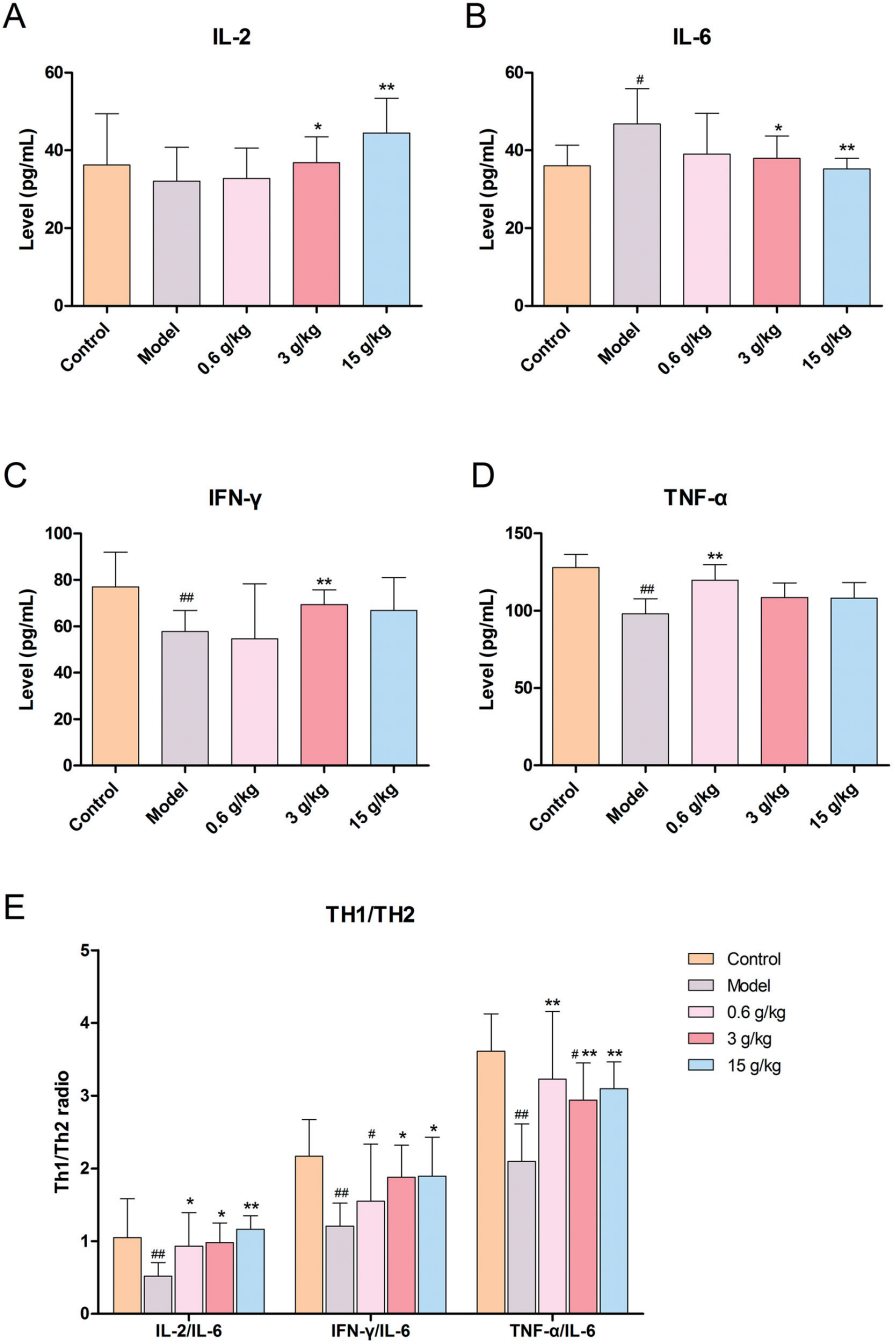
Effects of MLS on the levels of (A) interleukin-2 (IL-2) level, (B) interleukin-6 (IL-6), (C) interferon γ (IFN-γ), (D) tumour necrosis factor α (TNF-α) and (E) Th1/Th2 cell radio in RP rats (*n* = 10). ^#^*p* < 0.05 and ^##^*p* < 0.01 when compared with the control group; *p < 0.05 and **p < 0.01 when compared with the model group.

Compared with the control group, the model group exhibited significantly higher expression in the level of IL-6 (*p* < 0.05) and showed obvious lower expression in the levels of IFN-γ (*p* < 0.01) and TNF-α (*p* < 0.01). Compared with the model group, the different doses of MLS group had a recovery trend to the normal level in IL-2, IL-6, IFN-γ, and TNF-α. Especially, both of the medium and high dose MLS can significantly increase the level of IL-2 (*p* < 0.05; *p* < 0.01) and decrease the level of IL-6 (*p* < 0.05; *p* < 0.01) in RP rats, respectively. In addition, the medium dose MLS can significantly increase the level of IFN-γ (*p* < 0.01). Low dose MLS can significantly increase the level of TNF-α (*p* < 0.01) (Figure 2(A–D)). Compared with the control group, the ratio of Th1 and Th2 cells was significantly decreased in the model group. Each MLS administration group can obviously increase the Th1 and Th2 cells ratio in RP rats (Figure 2(E)).

### Metabonomics analysis

#### Method validation

**Figure 3.**
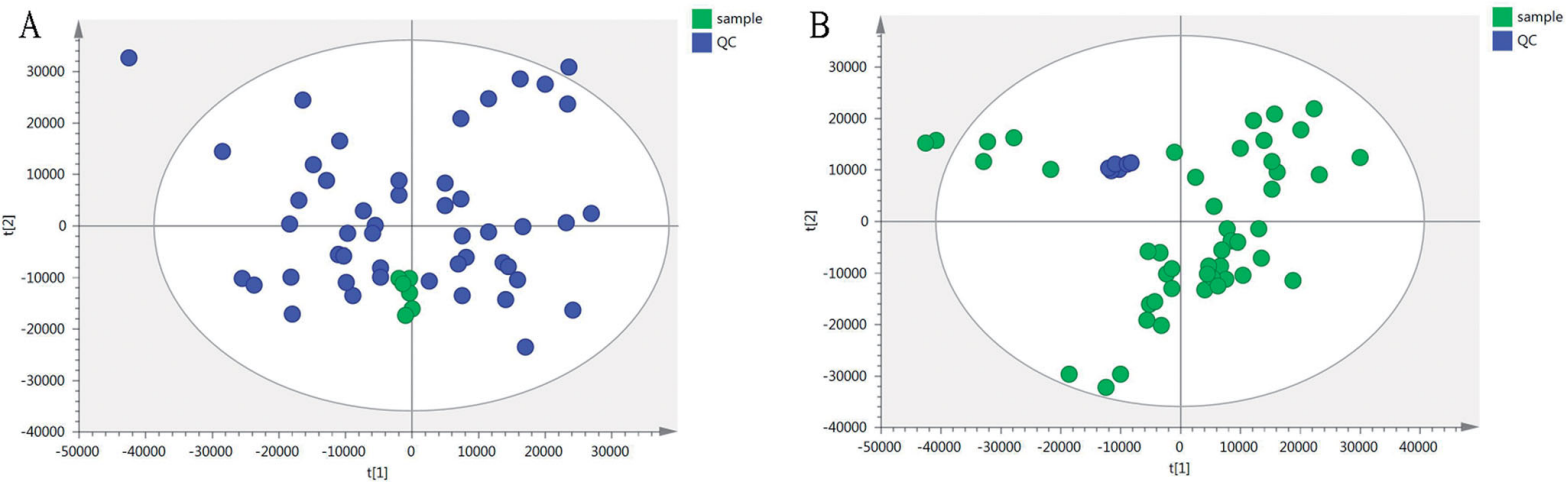
Principal component analysis (PCA) scores of plasma samples of all groups and quality control (QC) samples in (A) positive ion mode and (B) negative ion mode.

Metabonomics method was validated by clustering QC samples in the PCA model, the repeatability and stability of the system were investigated by monitoring the aggregation degree of QC samples. The results showed that QC samples gathered together and the degree of aggregation was good under positive and negative ion mode (Figure 3(A,B)), indicating that the repeatability and stability of the analysis method were good and the established method was suitable for batch metabolomics sample analysis.

#### Identification of potential biomarkers in RP rats and the metabolic pathway analysis

**Figure 4.**
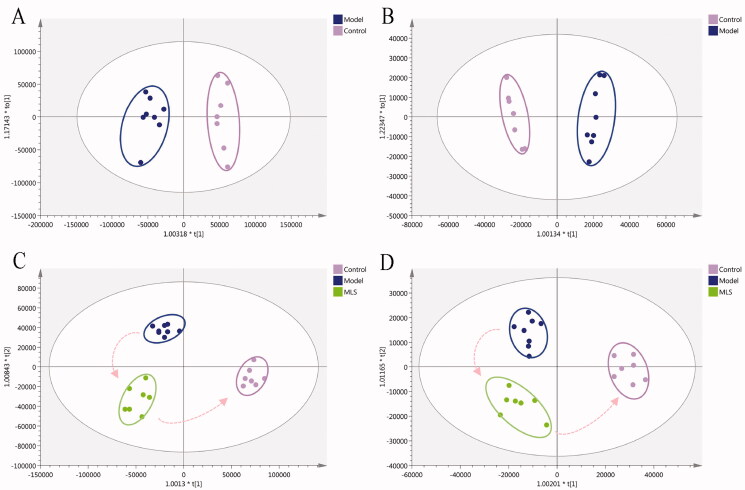
Orthogonal partial least squares discrimination analysis (OPLS-DA) scores of plasma samples of control group, model group, and MLS group: (A) control vs. model in positive ion mode; (B) control vs. model in negative ion mode; (C) control vs. model vs. MLS in positive ion mode; (D) control vs. model vs. MLS in negative ion mode.

**Figure 5.**
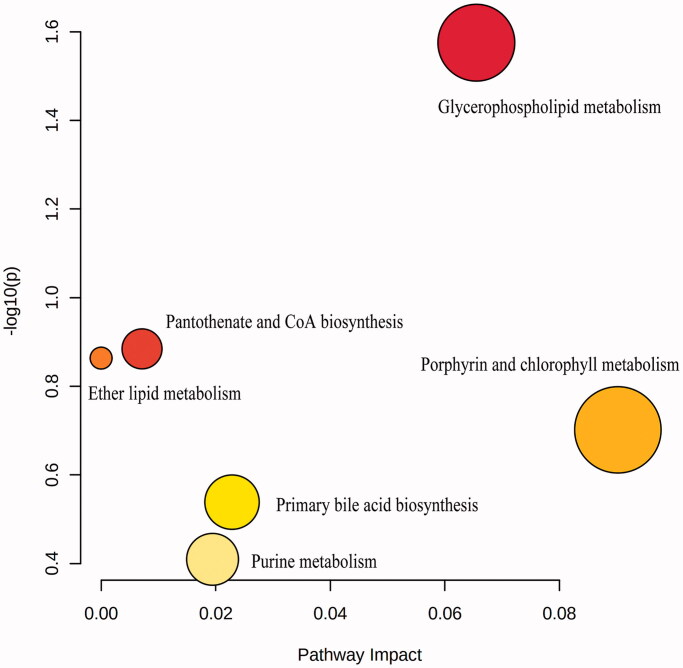
Metabolic pathway analysis of RP rats.

OPLS-DA was applied to explore potential biomarkers in RP rats compared with normal rats. As shown in Figure 4(A,B), the metabolites in the control and model groups were located in two different communities, which hinted the RP rats model was established successfully. Fifteen differential metabolites in plasma between RP rats and normal rats were obtained and identified by Progenesis QI software (VIP > 1, *p* < 0.05), as well as Lipidmaps, HMDB, KEGG, and Massbank (Table 1). According to KEGG, these potential biomarkers were related to 5 pathways of porphyrin and chlorophyll metabolism, glycerophospholipid metabolism, primary bile acid biosynthesis, purine metabolism, and pantothenate and CoA biosynthesis (Figure 5).

**Table 1.** Results of identified potential biomarkers in plasma via control vs model.

Compound name	HMDB ID	RT/ min	Formula	Observed *m/z*	Error/ ppm	Adduction	Trend	Metabolic pathways
l-α-Glycerylphosphorylcholine	0000086	1.62	C_8_H_20_NO_6_P	258.1099	−0.6575	[M + H]^+^	↓	Ether lipid metabolism; Glycerophospholipid metabolism
Pantothenic acid	0000210	2.69	C_9_H_17_NO_5_	220.1176	−1.5928	[M + H]^+^	↓	Pantothenate and CoA biosynthesis
Glycodeoxycholic acid	0000631	11.47	C_26_H_43_NO_5_	450.3208	−1.3535	[M + H]^+^	↓	–
LysoPC(18:3(9Z,12Z,15Z))	0010388	12.38	C_26_H_48_NO_7_P	518.3236	−0.9871	[M + H]^+^	↑	Glycerophospholipid metabolism
LysoPC(P-18:0)	0013122	14.93	C_26_H_54_NO_6_P	508.3760	−0.3047	[M + H]^+^	↓	Glycerophospholipid metabolism
LysoPC(20:1(11Z))	0010391	16.27	C_28_H_56_NO_7_P	550.3862	−0.9368	[M + H]^+^	↑	Glycerophospholipid metabolism
Protoporphyrin IX	0000241	18.18	C_34_H_34_N_4_O_4_	563.2645	−1.3101	[M + H]^+^	↓	Porphyrin and chlorophyll metabolism
Uric acid	0000289	2.11	C_5_H_4_N_4_O_3_	167.0203	−4.3337	[M-H]^-^	↑	Purine metabolism
Indolelactic acid	0000671	9.27	C_11_H_11_NO_3_	204.0663	−1.3372	[M-H]^-^	↑	–
Taurodeoxycholic acid	0000896	10.13	C_26_H_45_NO_6_S	498.2908	2.7170	[M-H]^-^	↑	–
Glycochenodeoxycholic acid	0000637	10.22	C_26_H_43_NO_5_	448.3079	2.4478	[M-H]^-^	↓	Primary bile acid biosynthesis
7-Ketodeoxycholic acid	0000391	11.16	C_24_H_38_O_5_	451.2711	1.0858	[M + Cl]^-^	↓	–
Isoursodeoxycholic acid	0000686	13.03	C_24_H_40_O_4_	391.2862	2.1947	[M-H]^-^	↓	–
LysoPC(22:5(7Z,10Z,13Z,16Z,19Z))	0010403	13.50	C_30_H_52_NO_7_P	614.3483	2.6375	[M + Cl]^-^	↑	Glycerophospholipid metabolism
LysoPC(22:4(7Z,10Z,13Z,16Z))	0010401	14.45	C_30_H_54_NO_7_P	616.3635	2.6792	[M + FA-H]^-^	↑	Glycerophospholipid metabolism

*Note*: ↑ and ↓ represent higher and lower expression in model group compared to control group.

#### Screening of potential biomarkers for MLS antiperspirant effect

**Figure 6.**
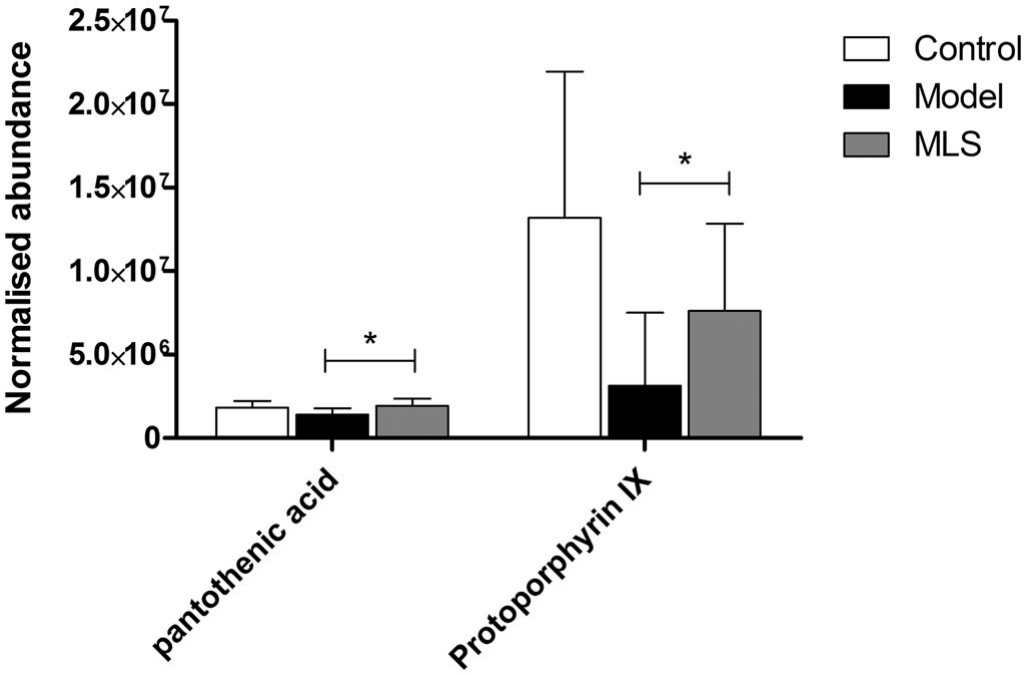
Metabolic regulation of MLS on RP rats. *p < 0.05 when compared with the model group.

The plasma metabolites data of the low dose MLS group was used to investigate its mechanism on antiperspirant effect. The results of OPLS-DA showed that different groups clustered in different areas. The MLS group was located between the control and model groups and had a visual movement trend from the model to the control, indicating that MLS was effective on RP rats (Figure 4(C,D)). Pantothenic acid and protoporphyrin IX were the two potential biomarkers in plasma that responded to the antiperspirant effect of MLS on RP rats (Figure 6), which related to the pathways of pantothenate and CoA biosynthesis and porphyrin metabolism.

## Discussion

In this study, the antiperspirant effect of MLS was comprehensively evaluated based on the RP rats. The RP rats showed mental depression, slow response, sluggishness, dry hair colour, and arched back, indicating that the animal model was successfully established in our study. It was found that low dose MLS had a noticeable effect on the decreasing of sweat points numbers in RP rats, revealing that MLS had a good antiperspirant effect. Low dose MLS showed a recovery trend in the thymus index regarding organ indexes. The thymus is the central immune organ of rats. It provides a complex and meticulous microenvironment for the growth and development of T cells, which is the place for the development and maturation of T cells (Xu et al. 2021). This indicated that MLS had improvement on immune function in RP rats.

T cells could regulate cell growth, differentiation, and cell-cell interaction by producing IL-2, IL-6, IFN-γ, TNF-α, and other cytokines (Wong et al. 2021). IL-2 could promote the proliferation and differentiation of lymphocytes and NK cells, and produce antibodies and cytokines such as IL-6 and TNF (Eaton-Fitch et al. 2021). IL-6 is a necessary cytokine for B cells for antibody production and T cell stimulation at different differentiation stages (Kim et al. 2021). IFN-γ can promote the killing activity of T cells, activate neutrophils, and promote the differentiation of CD8^+^ T cells into Th1 cells. TNF-α has a strong effect on the immune system and stimulates the immune and inflammatory response of the body (Li and Zeng 2021). IL-2, IL-6, IFN-γ, and TNF-α play an indispensable role in the immune response process and can comprehensively reflect the immune function of the whole body as an essential part of cytokines *in vivo*. In this study, MLS could promote the secretion of Th1 cytokines such as IL-2, IFN-γ, and TNF-α and inhibit the secretion of Th2 cytokines such as IL-6 in the serum of RP rats, indicating that MLS could restore immune function in RP rats by improving Th1/Th2 balance.

In plasma metabolomics, 15 potential biomarkers were found related to RP rats. The metabolic pathways mainly involved porphyrin and chlorophyll metabolism, glycerophospholipid metabolism, primary bile acid biosynthesis, purine metabolism, pantothenate and CoA biosynthesis, etc. MLS could reverse the levels of pantothenic acid and protoporphyrin IX in RP rats, indicating that the effect of MLS mainly involved the regulation of pantothenate and CoA biosynthesis, as well as porphyrin metabolism.

Pantothenic acid, also known as vitamin B5, is a precursor of CoA. Recently, the relationship between pantothenic acid and immunity has attracted broad attention. Pantothenic acid can promote immune cells to produce cytokines (He et al. 2018). It plays a significant role in inflammation and regulates the innate immune response by adjusting CoA levels (Jung et al. 2017). CoA mainly plays the function of acyl carrier in metabolism, participates in the decomposition of sugar, fat and protein, promotes energy metabolism, and scavenges oxygen free radicals (Depeint et al. 2006; Naquet et al. 2020). CoA also participates in important biochemical reactions in the body, such as the tricarboxylic acid cycle, fatty acid synthesis and oxidation, amino acid metabolism, etc. (Shedid et al. 2018). Therefore, the metabolic pathway of pantothenate and CoA biosynthesis plays a vital role in body physiological health. In this study, MLS can significantly reverse the level of pantothenic acid to normal expression, indicating that MLS had good regulation on pantothenate and CoA biosynthesis. After the improvement of pantothenate and CoA biosynthesis, RP rats' energy metabolism and immune function could be improved to improve pathological sweating.

Protoporphyrin IX is a critical metabolite in porphyrin metabolism, whose level is vital to the health of the body. It can promote cell tissue respiration and improve protein and glucose metabolism (Zhu et al. 2020). Protoporphyrin IX is the intermediate product in the process of haemachrome and a precursor of haemoglobin (Immenschuh et al. 2017). Abnormal porphyrin metabolism can lead to the accumulation of porphyrins *in vivo*, and then damage the function of mitochondria. Mitochondria can convert food and oxygen into adenosine triphosphate (ATP) through the respiration effect to meet the large energy needs of the body (Paes et al. 2020). In this study, MLS could significantly increase the level of protoporphyrin IX in RP rats, indicating that MLS could improve porphyrin metabolism and reduce porphyrin accumulation in RP rats to restore cell mitochondrial function and promote energy production. This is beneficial to improve sweating symptoms in RP rats.

On the whole, MLS showed a good antiperspirant effect on RP rats. The possible mechanism may be related to the improvement of pantothenate and CoA biosynthesis, as well as porphyrin metabolism. There was no doubt that this was a potential link, and more research was encouraged in the future. Additionally, there remained a limitation in our study. The potential biomarkers were putatively identified *via* various commercial databases in this study, needing further experimental verification.

## Conclusions

This study demonstrated that MLS had a good antiperspirant effect on RP rats and could regulate immune function by promoting the Th1/Th2 ratio balance. The effect mechanism of MLS may be related to the improvement of pantothenate and CoA biosynthesis, as well as porphyrin metabolism, which was beneficial to regulating immune function and promoting energy production. These findings might promote our understanding of the specific effect mechanism of MLS against RP rats and provide an experimental basis for the clinical application of MLS.
